# Clinical Characteristics and Outcomes of Hospital-Acquired Lower Gastrointestinal Bleeding: A Single Centre Retrospective Cohort Study

**DOI:** 10.7759/cureus.32651

**Published:** 2022-12-17

**Authors:** Hidehiro Someko, Haruhisa Shimura, Yasushi Tsujimoto, Yuji Okazaki, Toshiaki Shiojiri

**Affiliations:** 1 Internal Medicine, Asahi General Hospital, Asahi, JPN; 2 Gastroenterology and Hepatology, Asahi General Hospital, Asahi, JPN; 3 Department of Healthcare Epidemiology, Graduate School of Medicine and Public Health, Kyoto University, Kyoto, JPN; 4 Emergency Department, Hiroshima City Hiroshima Citizens Hospital, Hiroshima, JPN

**Keywords:** hospital-acquired complication, haemostatic endoscopic intervention, gastrointestinal tract, haematochezia, rebleeding, lower gastrointestinal bleeding

## Abstract

Background

Lower gastrointestinal bleeding (LGIB) is common in inpatient and outpatient settings; however, there are limited studies on the clinical characteristics and patient outcomes of those with hospital-acquired LGIB.

Methods

We performed a retrospective cohort study of patients with hospital-acquired LGIB who underwent colonoscopy during hospitalization between January 2017 and December 2021. We described the clinical characteristics, etiology, and clinical outcomes of patients stratified as those undergoing colonoscopy within 24 hours from haematochezia onset (early colonoscopy group) or after 24 hours from onset (late colonoscopy group). We used multivariable logistic regression to identify factors associated with endoscopic intervention in the early and late colonoscopy groups.

Results

Of the 272 patients included, the median age was 79 years (interquartile range: 72-85 years), 153 (56%) were bedridden, and 172 (63%) had hypoalbuminemia. The most frequent etiology was rectal ulcer (101 cases, 37%), whereas 7 (2.6%) had diverticular bleeding. The endoscopic intervention was performed on 16.7% and 7.9% of early and late colonoscopy patients. There were more patients with both non-severe and severe rebleeding in the early colonoscopy group (16% and 12%, respectively) than in the late colonoscopy group (11% and 6.5%, respectively). Colonoscopy-on-worktime was the only factor independently associated with a higher occurrence of endoscopic intervention.

Conclusions

In our sample, very old patients with hospital-acquired LGIB required endoscopy mainly due to rectal ulcers. Further studies will be necessary to investigate the differences between community-acquired LGIB and hospital-acquired LGIB and the optimal timing of colonoscopy for these patients.

## Introduction

Lower gastrointestinal bleeding (LGIB), which usually manifests as haematochezia, is defined as bleeding that originates from the gastrointestinal tract, distal to the ligament of Treitz. LGIB can have serious clinical outcomes, although the bleeding usually stops spontaneously [1,2]. The incidence of LGIB is estimated to be 20-126/100,000 in the outpatient population [3-6] and 330/100,000 in the inpatient population [7]. LGIB has a lower mortality rate than upper gastrointestinal bleeding [6,8]. However, rebleeding or readmission is not rare [9] and occasionally requires blood transfusion, hemostatic endoscopic intervention, radiological intervention, or surgery.

There are limited studies on LGIB that occurs after admission with symptoms other than haematochezia (hospital-acquired LGIB). Most previous studies focused on patients with LGIB who acquired the condition before hospitalization (community-acquired LGIB) [2,10-13]. Performing colonoscopy in patients with hospital-acquired lower gastrointestinal bleeding (LGIB) is more challenging than in patients with community-acquired LGIB because patients with hospital-acquired LGIB are older and likely to have more comorbidities than those with community-acquired LGIB [1,7]. The most common cause of hospital-acquired LGIB is rectal ulcers, and the most common cause of community-acquired LGIB is diverticular bleeding [1,7,14]. A recent systematic review found that early colonoscopy for community-acquired LGIB was not associated with reduced in-hospital mortality or rate of rebleeding [12]. However, it is unknown whether early colonoscopy for hospital-acquired LGIB could affect clinical outcomes such as in-hospital mortality, endoscopic hemostasis, or rebleeding. Factors associated with endoscopic hemostasis in community-acquired LGIB have been previously studied, and these factors have not been studied for hospital-acquired LGIB [14]. This information could help identify those patients with hospital-acquired LGIB who may benefit from early endoscopy.

The objectives of this study were to: describe the patient characteristics and the clinical outcomes of patients with hospital-acquired LGIB; compare findings between patients who underwent early colonoscopy (defined as ≤ 24 hours from the recognition of haematochezia) and late colonoscopy (> 24 hours from the recognition of haematochezia), and; to examine factors associated with endoscopic intervention in hospital-acquired LGIB.

## Materials and methods

Study design and settings

This single-center, retrospective descriptive study was conducted at Asahi General Hospital (a teaching hospital located in a suburban area of Japan) between January 2017 and December 2021. This study was approved by the Institutional Ethical Committee (approval no: 2022071918) of Asahi General Hospital and is in concordance with the Strengthening the Reporting of Observational Studies in Epidemiology (STROBE) guidelines [15].

Patient selection

The colonoscopy records of patients hospitalized during the study were collected and assessed for eligibility. Patients who fulfilled the following criteria were included: 1) aged 18 years or above, 2) had haematochezia which started at any time after hospitalization, and 3) underwent colonoscopy for haematochezia. The exclusion criteria were as follows: 1) patients who had haematochezia on admission, 2) patients who had haematochezia onset in the intensive care unit, 3) patients who had upper gastrointestinal bleeding confirmed by upper endoscopy, and 4) patients who had haematochezia following lower endoscopic intervention (e.g., endoscopic mucosal resection).

Definitions

Haematochezia was defined as dark or bright red blood, with or without clots per rectum; maroon-colored or blood-mixed stool; or melaena without haematemesis [16]. The patient was considered bedridden if the Eastern Cooperative Oncology Group performance status score was 4 [17]. Worktime was defined as 08:30 - 17:00 on weekdays, excluding national holidays. Non-worktime was defined as any time outside of the worktime parameters. Early colonoscopy and late colonoscopy were defined as a colonoscopy performed within 24 hours or after 24 hours of haematochezia recognition, respectively [18]. A complete colonoscopy was defined as a colonoscopy with intubation of the caecum. Rebleeding was defined as prolonged or recurrent haematochezia after the first colonoscopy. Severe rebleeding was defined as rebleeding with two or more units of packed red blood cells (PRBC) transfused or a 20% decrease in hematocrit [19].

Data collection

We collected the data of eligible patients from either direct confirmation or the data warehouse for the electronic medical records of the institution. The following data were collected through direct confirmation: the date and time of the first haematochezia episode, as recognized by a doctor or nurse; whether the colonoscopy was performed within 24 hours of the recognition of haematochezia; past medical history; comorbidities; the symptoms of haematochezia; vital signs; physical examination; performance status on haematochezia recognition; findings of contrast-enhanced computed tomography (CT), performed after haematochezia recognition and before colonoscopy; type of bowel preparation; and findings of colonoscopy, including intervention. The date and time of the first haematochezia diagnosis were classified as worktime or non-worktime. Comorbidities were evaluated using the Charlson Comorbidity Index [20]. If symptoms, vital signs, or physical examination results were not recorded at the time of haematochezia recognition, they were regarded as missing. The following data were also collected from the data warehouse: the department where haematochezia was recognized, the date and time of colonoscopy, laboratory data on haematochezia recognition, and prescribed medications within one week before haematochezia.

Outcomes

Outcomes were evaluated using data from electronic medical records. The underlying cause of the haematochezia was diagnosed using relevant clinical information; findings of contrast-enhanced CT; or first and follow-up colonoscopy and upper endoscopy, if available. The number of units of PRBC that were transfused between haematochezia recognition and the day after colonoscopy was recorded. Whether in-hospital death was related to LGIB was determined based on available clinical information.

Statistical analysis

We tabulated the baseline characteristics and outcomes, stratified according to early or late colonoscopy. We also showed the number of patients with available data when there were any patients with missing values in the variables. Factors associated with the endoscopic intervention were investigated using univariate and multivariate logistic regression analyses. All patients of LGIB fulfilling inclusion/exclusion criteria from January 2017 to December 2021 were included. We reported the crude odds ratio (OR) with a 95% confidence interval (CI) and adjusted the OR with a 95% CI. Continuous data are shown as medians (interquartile range) unless otherwise indicated. Dichotomous data are presented as numbers (percentages). All descriptive and statistical analyses were performed using R 4.2.3, using the gtsummary package [21].

## Results

Patient characteristics

**Figure 1:**
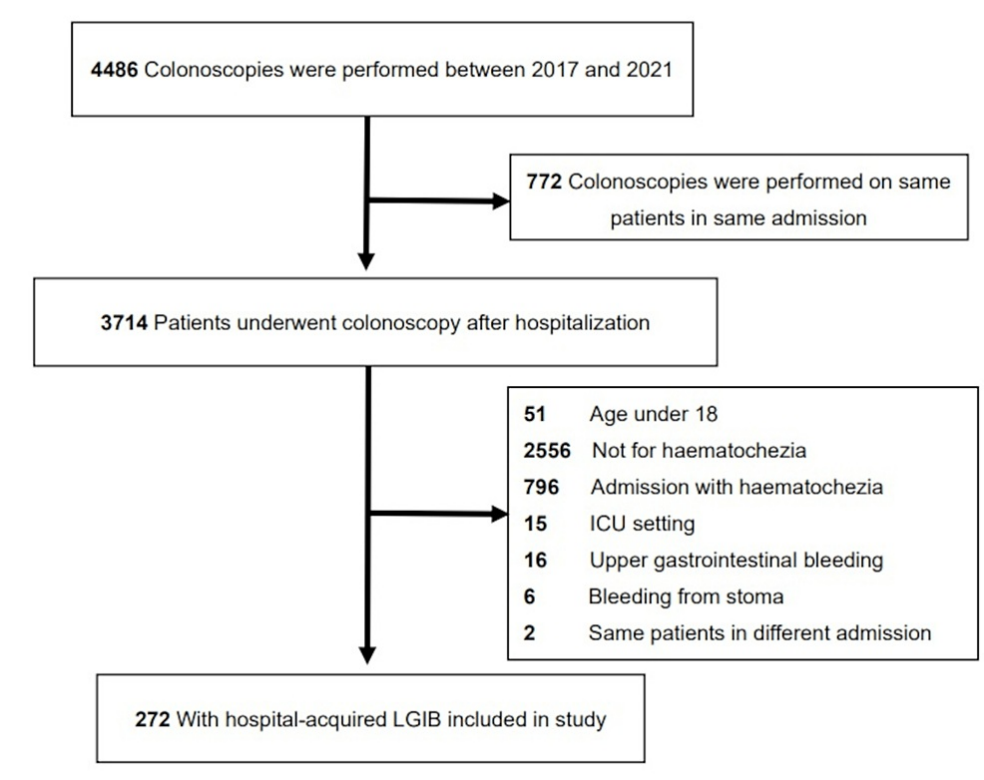
Patient selection flow chart ICU intensive care unit, LGIB lower gastrointestinal bleeding

During the study period, 4486 colonoscopy records were obtained. After excluding duplicate records and patients who met the exclusion criteria, 272 patients were included in the study (Figure 1).

The patient demographics are shown in Table 1. There were 134 patients in the early colonoscopy group and 138 in the late colonoscopy group. The median ages were 80 (72-86) and 78 (70-84), respectively. In both groups, most episodes of haematochezia were found during worktime (90% overall), and 56% of patients were bedridden. The median systolic blood pressure was 113 (100-132) mmHg in the early colonoscopy group and 119 (103-141) mmHg in the late colonoscopy group. There were more hypotensive patients (systolic pressure ≤ 90 mmHg) in the early colonoscopy group than in the late colonoscopy group (7.8% vs. 4.3%, respectively). The median pulse rate was 83 (70-96) beats per minute (bpm) for the early colonoscopy group and 83 (73-94) bpm for the late colonoscopy group. The median hemoglobin was 10.4 g/dL (8.2-12) for the early colonoscopy group and 10g/dL (8.27-11.88) for the late colonoscopy group. There was no significant difference in the distribution of hemoglobin concentrations between the groups. The median serum albumin levels were 2.5 (2.1-2.92) g/dL in the early colonoscopy group and 2.65 (2.2-3.2) g/dL in the late colonoscopy group. There were more patients with hypoalbuminemia in the early colonoscopy group than in the late colonoscopy group (75% vs. 60%, respectively). The Median Charlson Comorbidity Index score was 2 in both groups, although there were more patients with hemiplegia, cerebrovascular disease, and dementia in the early colonoscopy group. No bowel preparation was used for 42% of the patients in the early colonoscopy group, whereas polyethylene glycol was used for 49% of the patients in the late colonoscopy group. Antithrombotic medications, including aspirin, were used equally in both groups.

**Table 1:** Baseline characteristics of the patients ^a ^Number in square brackets shows the number of patients with available data ^b ^Worktime was defined as 8:30-17:00 on weekdays. Non-worktime was defined as otherwise ^c ^Patients with performance status 4 were defined as bedridden ^d^ Neuropathy, retinopathy, or renal impairment was defined as diabetic complication SBP systolic blood pressure;, DRE digital rectal exam;, LGIB lower gastrointestinal bleeding;, COPD chronic obstructive disease;, CT computed tomography;, NSAIDs nonsteroidal anti-inflammatory agents, COX-2 cyclooxygenase-2

Characteristics	Timing of colonoscopy^a^	
	Early, N = 134	Late, N = 138
Time to colonoscopy, hours	7 (4, 13)	66 (32, 123)
Age, years, ≥ 70, n (%)	114 (85%)	107 (78%)
Male, n (%)	77 (57%)	76 (55%)
Admission in Department of Gastroenterology, n (%)	13 (9.7%)	6 (4.3%)
Haematochezia on non-worktime^b^, n (%)	118 (88%)	126 (91%)
Colonoscoy on non-worktime, n (%)	53 (40%)	22 (16%)
Bedridden^c^, n (%)	85 (63%)	68 (49%)
Systolic blood pressure (mmHg)	{128}	{116}
≥ 160	5 (3.9%)	11 (9.5%)
130-159	36 (28%)	36 (31%)
120-129	14 (11%)	10 (8.6%)
90-119	63 (49%)	54 (47%)
<90	10 (7.8%)	5 (4.3%)
Pulse rate (/minutes)	{127}	{113}
<69	31 (24%)	22 (19%)
70-89	50 (39%)	52 (46%)
90-109	36 (28%)	32 (28%)
≥ 110	10 (7.9%)	7 (6.2%)
Diarrhea, n (%)	3 (2.2%)	10 (7.2%)
Syncope,n (%)	2 (1.5%)	0 (0%)
Abdominal tenderness, n (%)	4 (3.1%) {130}	8 (6.1%) {132}
Blood on DRE, n(%)	64 (74%) {86}	42 (58%) {72}
Haemoglobin (g/dl), n (%)	{129}	{134}
≥ 13	14 (11%)	24 (18%)
11-12.9	41 (32%)	25 (19%)
9-10.9	34 (26%)	45 (34%)
7-8.9	28 (22%)	29 (22%)
<7	12 (9.3%)	11 (8.2%)
Albumin < 3g/dl, n (%)	93 (75%) {124}	77 (60%) {128}
Past history of LGIB, n (%)	7 (5.2%)	8 (5.8%)
Diabetes mellitus		
None or only diet	95 (71%)	99 (72%)
Without complication^d^	21 (16%)	14 (10%)
With complication	18 (13%)	25 (18%)
Hemiplegia, n (%)	31 (23%)	22 (16%)
Cerebrovascular disease, n (%)	37 (28%)	27 (20%)
Dementia, n (%)	13 (9.7%)	8 (5.8%)
COPD, n (%)	13 (9.7%)	9 (6.5%)
Collagenous disease, n (%)	8 (6.0%)	5 (3.6%)
Peptic ulcer, n (%)	12 (9.0%)	12 (8.7%)
Myocardial infarction, n (%)	30 (22%)	29 (21%)
Congestive heart failure, n (%)	9 (6.7%)	16 (12%)
Peripheral artery disease, n (%)	14 (10%)	18 (13%)
Leukemia, n (%)	0 (0%)	1 (0.7%)
Lymphoma, n (%)	0 (0%)	4 (2.9%)
Cancer, n (%)		
None	112 (84%)	116 (84%)
Localized	7 (5.2%)	11 (8.0%)
Metastatic	15 (11%)	11 (8.0%)
Liver disease, n (%)	8 (6.0%)	6 (4.3%)
Chronic kidney disease, n (%)		
None	90 (67%)	98 (71%)
Without hemodialysis	30 (22%)	28 (20%)
With hemodialysis	14 (10%)	12 (8.7%)
Charlson comorbidity index	2.00 (1.00, 4.00)	2.00 (1.00, 4.00)
CT with enhancement, n (%)	53 (40%)	48 (35%)
Bowel preparation, n (%)	{133}	{136}
None	56 (42%)	23 (17%)
Glycerin enema	64 (48%)	46 (34%)
Polyethylene glycol	13 (9.8%)	67 (49%)
NSAIDs, n (%)	13 (9.7%)	7 (5.1%)
COX 2 inhibitor, n (%)	6 (4.5%)	8 (5.8%)
Aspirin, n (%)	46 (34%)	43 (31%)
Antiplatelet other than aspirin, n (%)	29 (22%)	28 (20%)
Anticoaglant, n (%)	56 (42%)	55 (40%)
Steroid, n (%)	19 (14%)	28 (20%)

Outcomes

At the time of data collection, all patients included in this study had been discharged, and no missing data were identified (Table 2). In the early colonoscopy group, 50% of the patients were diagnosed with rectal ulcers, compared to 25% in the late colonoscopy group. Diverticular bleeding was diagnosed in less than 5% of patients in both groups. The diagnosis was unknown in 13% of the early and 20% of the late colonoscopy group. The endoscopic intervention was performed in 16.7% and 7.9% of early and late colonoscopy patients. There were more non-severe and severe bleeding patients in the early colonoscopy group (16% and 11%, respectively) than in the late colonoscopy group (12% and 6.5%, respectively). LGIB-related in-hospital death occurred in two cases, both in the late colonoscopy group.

**Table 2:** Outcomes of hospital -acquired lower gastrointestinal bleeding ^a^ Rectal ulcer includes 12 cases of rectal erosion ^b ^Other diagnosis includes: Radiation colitis, colorectal varix, inflammatory bowel disease ^c^ Severe rebleeding was defined as rebleeding which needs ≥2 PRBC transfusions or a 20% decrease in haematocrit. PRBC packed red blood cell

	Timing of colonoscopy	
Outcomes	Early, N = 134^1^	Late, N = 138^1^
Complete colonoscopy, n (%)	16 (12%)	61 (44%)
Diagnosis		
Rectal ulcer^a^	67 (50%)	35 (25%)
Hemorrhoid bleeding	10 (7.5%)	15 (11%)
Colorectal cancer	4 (3.0%)	13 (9.4%)
Ischemic colitis	8 (6.0%)	7 (5.1%)
Non-specific colitis	3 (2.2%)	10 (7.2%)
Polyp	6 (4.5%)	5 (3.6%)
Angioectasia	2 (1.5%)	7 (5.1%)
Anal lesion	4 (3.0%)	4 (2.9%)
Diverticular bleeding	5 (3.7%)	3 (2.2%)
Small bowel bleeding	1 (0.7%)	5 (3.6%)
Infectious colitis	2 (1.5%)	2 (1.4%)
Postoperative astomotic bleeding	1 (0.7%)	2 (1.4%)
Metastatic tumor	0 (0%)	2 (1.4%)
Other diagnosis^b^	3 (2.2%)	0 (0%)
Unknown	18 (13%)	28 (20%)
Stigmata of recent hemorrhage, n (%)	26 (19%)	14 (10%)
Transfusion of PRBC, units, n (%)		
2	29 (22%)	27 (20%)
4	20 (15%)	17 (13%)
≥6	0 (0%)	0 (0%)
Endoscopic intervention, n (%)		
Clipping	22 (16%)	10 (7.2%)
Argon plasma coagulation	1 (0.7%)	1 (0.7%)
Surgery, n (%)	0 (0%)	1 (0.7%)
Interventional radiology, n (%)	0 (0%)	1 (0.7%)
Rebleeding, n (%)	21 (16%)	15 (11%)
Severe^c^	16 (12%)	9 (6.5%)
Inhospital death, n (%)		
Not related	36 (100%)	22 (92%)
Related	0 (0%)	2 (8.3%)
^1^n (%)		

Factors associated with endoscopic intervention

No patients with liver disease or collagenous vascular disease underwent endoscopic intervention; these variables could not be used in univariable and multivariable analyses with a logistic regression model (Table 3). Admission to the Department of Gastroenterology and congestive heart failure was not used in the multivariable analysis due to the low number of patients who underwent endoscopic intervention. Age ≥ 70 years and colonoscopy at work time were the only factors associated with an endoscopic intervention.

**Table 3:** Factors associated with endoscopic intervention ^1^OR = Odds Ratio, CI = Confidence Interval SBP systolic blood pressure;, DRE digital rectal exam;, LGIB lower gastrointestinal bleeding;, COPD chronic obstructive disease;, CT computed tomography;, NSAIDs nonsteroidal anti-inflammatory agents, COX-2 cyclooxygenase-2

Factors	Univariable		Multivariable			
	OR^1^	95% CI^1^		OR^1^	95% CI^1^	
Age 70 or above	4.15	1.20, 26.1		5.01	0.95, 41.8	
Male	0.50	0.24, 1.03		0.79	0.25, 2.44	
Admission to the department of gastroenterology	0.37	0.02, 1.89				
Hematochezia on work time	0.62	0.23, 1.96		0.26	0.04, 1.78	
Colonoscopy on work time	5.47	2.60, 11.9		13.5	4.54, 47.8	
Bedridden	1.30	0.63, 2.77		1.03	0.30, 3.71	
Pulse rate						
≦69	—	—		—	—	
70-89	1.35	0.51, 4.00		1.02	0.22, 5.34	
90-109	1.04	0.34, 3.37		1.58	0.30, 9.39	
110≧	1.68	0.32, 7.28		6.62	0.74, 61.4	
Systolic blood pressure						
160≧	—	—		—	—	
130-159	1.26	0.29, 8.75		2.80	0.27, 42.2	
120-129	1.40	0.24, 11.1		3.45	0.23, 74.8	
90-119	0.88	0.21, 5.97		0.73	0.07, 10.1	
≦89	1.08	0.12, 10.1		2.30	0.11, 52.2	
Hemoglobin						
13≧	—	—		—	—	
11-12.9	4.00	1.01, 26.7		5.69	0.85, 57.6	
9-10.9	2.91	0.73, 19.5		2.90	0.42, 28.2	
7-8.9	2.52	0.57, 17.6		4.40	0.49, 54.5	
<7	0.82	0.04, 9.03		0.47	0.01, 9.84	
Albumin < 3 g/dl	1.21	0.54, 2.88		0.76	0.21, 2.81	
History of LGIB	1.08	0.16, 4.16		1.20	0.10, 8.58	
Diabetes mellitus						
None or only diet	—	—		—	—	
Without complication	1.69	0.63, 4.11		3.32	0.84, 13.2	
With complication	0.33	0.05, 1.17		0.80	0.07, 5.60	
Hemiplegia	0.51	0.15, 1.38		0.46	0.08, 2.22	
Cerebrovascular disease	1.20	0.50, 2.64		1.07	0.32, 3.40	
Dementia	1.18	0.27, 3.75		0.61	0.09, 3.37	
COPD	1.12	0.25, 3.52		0.71	0.07, 4.75	
Myocardial infarction	0.75	0.27, 1.79		1.06	0.17, 5.94	
Congestive heart failure	0.58	0.09, 2.11				
Peripheral artery disease	0.43	0.07, 1.53		0.41	0.04, 2.52	
Cancer						
None	—	—		—	—	
Localized	0.83	0.13, 3.10		2.14	0.23, 16.1	
Metastatic	0.55	0.09, 1.99		0.17	0.01, 1.52	
Chronic kidney disease						
None	—	—		—	—	
Without hemodialysis	1.04	0.42, 2.37		0.93	0.26, 3.07	
With hemodialysis	0.26	0.01, 1.32		0.36	0.01, 6.12	
NSAIDs	1.26	0.28, 4.02		1.00	0.14, 5.89	
Aspirin	0.84	0.37, 1.79		0.75	0.17, 3.01	
Antithrombotics	1.02	0.49, 2.11		0.54	0.16, 1.74	
Steroid	1.03	0.37, 2.50		2.18	0.54, 8.38	

## Discussion

In this retrospective analysis of 272 patients with hospital-acquired LGIB who underwent colonoscopy during hospitalization, we found that rectal ulcers were the most common cause of bleeding. Patients who underwent early colonoscopy received more endoscopic interventions and were more likely to develop rebleeding. Our exploratory data analysis showed that colonoscopy during worktime was independently associated with a higher occurrence of endoscopic interventions.

Hospital-acquired LGIB may have clinical features different from those of community-acquired LGIB. In previous cohort studies, diverticular bleeding was the most common cause of community-acquired LGIB, occurring in 23-63% of cases [1,2,4,11,22]. However, the incidence of rectal ulcers was less than 10% in this population. Our results showed that rectal ulcers were the most frequent cause of hospital-acquired LGIB (overall 38%), and diverticular bleeding was low (overall 3%), which was consistent with previous studies of hospital-acquired LGIB [7,14]. The difference in clinical characteristics of inpatients vs. outpatients may explain the variation in the underlying cause of LGIB. In our cohort, 56% of the patients were bedridden, and 63% had malnutrition. The median serum albumin levels were 2.6 g/dL. Meanwhile, in the community-acquired LGIB cohort, fewer patients were bedridden or malnourished. For example, in a multicentre study conducted in Japan, only 2.4% of the patients were bedridden [11]. In that study, the median value of serum albumin in the cohort was 3.3 g/dL, which suggests that there were fewer patients with malnutrition than in our cohort. Malnutrition and impaired activity of daily living (ADL), which are known risk factors for rectal ulcers [23,24], may explain why rectal ulcers were the most common cause of haematochezia in hospitalized patients.

The outcomes of early colonoscopy for hospital-acquired LGIB may be similar to those of community-acquired LGIB. A systematic review of randomized controlled trials and observational studies of early vs. late colonoscopy for community-acquired LGIB demonstrated that rebleeding, endoscopic intervention, and identifying the bleeding source were more common in the early colonoscopy group. However, these findings were not statistically significant [12]. Our results were consistent with these findings, although patients with hospital-acquired LGIB have different clinical features. There are several reasons why early colonoscopy might not reduce rebleeding in hospital-acquired LGIB. First, the risk factors for rectal ulcers, such as malnutrition and impaired ADL, are challenging to resolve quickly. Second, more than half of the patients were taking antithrombotic medication, and the effects of antithrombotic medication can persist after discontinuation for days to weeks. Given these findings, whether early colonoscopy reduces rebleeding in patients with hospital-acquired LGIB remains in question.

The only factor we identified in univariable and multivariable analyses associated with endoscopic intervention was colonoscopy during work time. This may be due to the increased availability of staff required during an endoscopic procedure. A previous study built a model to predict outcomes, including endoscopic intervention, for community-acquired LGIB, with variables such as age, sex, systolic blood pressure, heart rate, and hemoglobin [17]. We could not compare our findings with this study as the outcomes measured in their study differed from ours. Further studies are needed to predict the outcomes of hospital-acquired LGIB.

Despite these limitations, our study is the first to describe the characteristics of patients with hospital-acquired LGIB and the clinical outcomes of colonoscopy in this population. We followed pre-specified protocols and standard reporting guidelines [15].

This study had several limitations. First, we could not collect data on patients with haematochezia who did not undergo colonoscopy because these patients could not be identified in the electronic medical records. Furthermore, our study only examined Japanese patients from a single-center location, many of whom were bedridden and suffering from malnutrition. Therefore, our results may not apply to all patients with hospital-acquired haematochezia. Patient demographics and clinical judgment (regarding the rationale for colonoscopy) may differ in other institutions. We could not perform statistical analyses on patient outcomes due to our small sample size and unequal data sets between early and late colonoscopy groups. However, to the best of our knowledge, our cohort study was the largest thus far that sought to investigate the clinical features and outcomes of hospital-acquired LGIB. Lastly, we could not use similar definitions in our study to those used in studies on community-acquired LGIB. For example, early colonoscopy in community-acquired LGIB studies is done from the time of admission or presentation to the emergency department. Our study measured early colonoscopy from the time of recognition of haematochezia. Given these limitations, studies with larger cohorts are necessary to investigate the factors associated with endoscopic intervention to identify patients who may benefit from an early colonoscopy. In addition, randomized controlled trials are needed to confirm that early colonoscopy does not improve the outcomes of patients with hospital-acquired LGIB.

## Conclusions

In detail, we described the clinical characteristics, etiology, and outcomes of hospital-acquired LGIB. In our study, very old patients with hospital-acquired LGIB underwent endoscopy mainly due to rectal ulcers. Although patients with hospital-acquired LGIB may have different clinical features from those with community-acquired LGIB, the outcomes of early colonoscopy may be similar to those of community-acquired LGIB. We recommend that doctors be aware of the difference in clinical features for community-acquired LGIB vs. hospital-acquired LGIB and use this information when considering early colonoscopy for their patients. Further studies, including prospective large cohort designs, are needed to investigate the factors associated with endoscopic intervention to identify patients who may benefit from an early colonoscopy. Randomized controlled trials are required to assess the efficacy of early colonoscopy for hospital-acquired LGIB.
